# Towards the Sustainability of the Plastic Industry through Biopolymers: Properties and Potential Applications to the Textiles World

**DOI:** 10.3390/polym14040692

**Published:** 2022-02-11

**Authors:** Antonella Patti, Domenico Acierno

**Affiliations:** 1 Department of Civil Engineering and Architecture (DICAr), University of Catania, Viale Andrea Doria 6, 95125 Catania, Italy; 2 CRdC Nuove Tecnologie per le Attività Produttive Scarl, Via Nuova Agnano 11, 80125 Naples, Italy

**Keywords:** textiles, sustainability, biopolymers

## Abstract

This study aims to provide an overview of the latest research studies on the use of biopolymers in various textile processes, from spinning processes to dyeing and finishing treatment, proposed as a possible solution to reduce the environmental impact of the textile industry. Recently, awareness of various polluting aspects of textile production, based on petroleum derivatives, has grown significantly. Environmental issues resulting from greenhouse gas emissions, and waste accumulation in nature and landfills, have pushed research activities toward more sustainable, low-impact alternatives. Polymers derived from renewable resources and/or with biodegradable characteristics were investigated as follows: (i) as constituent materials in yarn production, in view of their superior ability to be decomposed compared with common synthetic petroleum-derived plastics, positive antibacterial activities, good breathability, and mechanical properties; (ii) in textile finishing to act as biological catalysts; (iii) to impart specific functional properties to treated textiles; (iv) in 3D printing technologies on fabric surfaces to replace traditionally more pollutive dye-based and inkjet printing; and (v) in the implants for the treatment of dye-contaminated water. Finally, current projects led by well-known companies on the development of new materials for the textile market are presented.

## 1. Introduction

Currently, textile production is constantly expanding by catering to fashion, style, and marketing needs, as well as increasingly competitive technical challenges [1]. The prospect of innovation and development contrasts, however, with the high pollution of the environment caused by this industrial activity [2]. Between 1975 and 2018, the production of chemical and textile fibers nearly quadrupled, from 23.94 million metric tons to 105.6 million metric tons: a more than fourfold increase in less than 40 years [3]. Concerns about the environmental impact of fiber production and subsequent disposal operations have grown in prominence as demand for fabrics has increased. Several recent studies show that the textile industry endangers freshwater and atmosphere micro-systems due to the use of industrially harmful and toxic chemicals during the manufacturing process, as well as the release of these chemicals [4,5,6]. Inadequate collection and careless disposal of solid waste pollutes the land and air, endangering human health and the environment. As a result, countries should devote a significant portion of their effort to waste management [7].

In this context, emphasis was placed on the development of alternative eco-friendly solutions, green technologies, and sustainable manufacturing processes in order to avoid risks to human health while also limiting environmental issues. 

In our previous work [8], attention was paid to recent scientific research activities promoting the sustainability requirements of textile production and circular economy actions through the recovery of textile waste products and recycling fibers into composite materials. However, bio-based materials, i.e., a broad class of organic constituents produced from renewable resources endowed with specific structural and functional characteristics such as biodegradability, composability, or biocompatibility, could offer a potential solution to replace the conventional plastics (primarily made from polyester (PET) [9,10], polyamide (PA), and polypropylene (PP) [11,12,13]) involved in fabric and yarn production [14]. Biopolymers can be used in the textile industry as the constituent base material for the production of filaments and yarns, or to replace harmful chemicals in pre-treatment and finishing operations by providing textile substates with various potential functionalities, such as antibacterial and flame-retardant activity, UV protection, electric conductivity, and hydrophobicity [15]. In this study, we aimed to collect the most recent studies on prospective biopolymers in the development of textile production by demonstrating the potential of these materials in replacing common oil-derived plastics.

## 2. Background

Several applications of plastic-based materials exist today, involving not only commercial and industrial fields, but also everyday life products. The global production of plastics is continuously increasing due to different intrinsic properties, such as lightweight, low-cost, durable, and chemical resistant properties. In addition, the increase in the human population, together with rapid economic growth, continuous urbanization, and lifestyle changes are other factors considered responsible for the growing demand for plastic products [2].

However, the main drawback that should be considered in the widespread employment of plastic consists in the difficulty of properly disposing of plastic at the end of its life cycle, and its consequent accumulation in nature. It has been predicted that millions of tons of plastics will be released into the surroundings in the coming years [16]. Every year, tons of plastic waste accumulate in the oceans, including microplastics (1 to 5000 μm particles), seriously altering the marine environment and the lives of its inhabitants [17]. 

According to Jambeck et al. (2015) [18], 4.8 to 12.7 million tons of plastic debris enter the ocean each year, with cumulative inputs expected to increase tenfold by 2025. 

**Figure 1:**
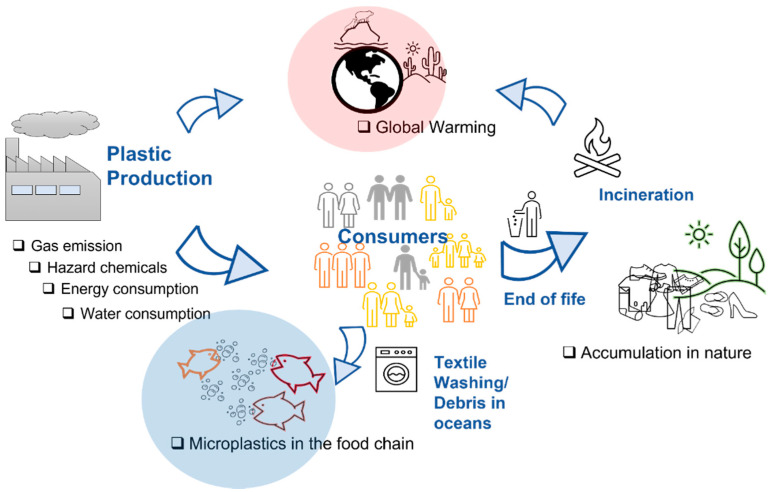
Environmental impact of plastics (black points) and corresponding sources (blue elements).

Furthermore, millions of tons of CO_2_ will be emitted in the atmosphere generated both from production processes and also from the final incineration at the end of the products’ life cycle [19]. The huge emission of greenhouse gases such as CO_2_ further contribute to global warming by leading to dramatic and irreversible weather changes. As a result of the industrial revolution, the CO_2_ concentration rose to over 390 ppm, and the global temperature rose by 0.9 °C. In 2019, the former rose to over 400 ppm, while the latter rose by 1.0 °C. Based on current trends, it is possible to imagine these levels exceeding 450 ppm and 1.5 °C in the next 20–40 years [20]. Environmental impact deriving from production, use, consumption, and end of life of plastics, is illustrated in Figure 1.

At the end of 2019, with the spread of the severe acute respiratory infection syndrome coronavirus 2 (SARS-CoV-2) pandemic, also known as COVID-19, which has affected millions of people worldwide, hundreds of countries, and thousands of victims, the global economy was influenced in three ways: directly, through production and demand, or supply chain and market disruption; and indirectly, through its financial impact on firms and financial markets [21]. As illustrated in a very recent paper by Mittal et al. [22], with the closing of shops and restaurants during lockdowns, people’s living conditions have been changed, causing concerns over waste generation routines. Plastic wastes have increased due to the extensive use of face masks, vaccine containers, packaging from food, medical industries, and online purchases. In order to reduce the cumulative effects, the substitution of petroleum-based plastics with bio-based plastics has also been presented. Data and hypothetical calculations on the amplified amount of plastic waste generated during COVID-19 have been collected in the work of Shams et al. [23].

The textile, fashion, and style industries were hit particularly hard by the pandemic’s spread and the associated containment measures. Shahbandeh (2021) [24] reported a drastic decrease in European textile production (equal to 26.9%) compared to the same period in 2019 (April and June 2020), when coronavirus cases reached a global peak. However, a remarkable increase in extra-EU textile trade of 154.2% was also confirmed. This was attributed to the import of personal protective equipment (PPE) that was required during Europe’s coronavirus crisis. Face masks and isolation gowns were identified as the first weapons of contagion protection in 2020. Authorities all over the world have encouraged, and in some cases mandated, the use of face masks in public places, and every adult on the planet has worn one. 

Face masks and gowns are mostly made from polypropylene given the versatile nature of this polymer, which allows thermal processes in different ways and forms, and the quick production on a large scale from monomers via chain-growth polymerization [25]. 

However, the unpredictability of the pandemic left the municipal solid waste (MSW) sector unprepared, even in countries with a longer tradition of sustainable waste management. Contrary to expectations, single-use masks and gloves have little impact on waste management, accounting for only 1% of the residual municipal solid waste collected annually. On the other hand, the dispersal of abandoned masks and gloves outside of indoor environments is causing environmental issues [26]. 

## 3. Biopolymers

Biopolymers can be produced from vegetal or animal-derived polysaccharides (i.e., starch, cellulose, lignin), proteins (i.e., chitosan, collagen) and lipids (i.e., wax, fatty acids), bacterial activities (i.e., polyhydroxyalkanoates (PHA)), the conventional synthesis of bio-derived monomers (i.e., polylactides (PLA) and polyglycolide (PLG)) or synthetic monomers (i.e., polycaprolactone (PCL)s, polyester-amides (PEA), an aliphatic or aromatic co-polyester (PBSA, PBAT)) [27].

The term “bio-based” does not necessarily imply “biodegradable” [28]. According to ASTM D6400-99 [29], a biodegradable plastic is a degradable plastic in which the degradation results from the action of naturally occurring microorganisms, such as bacteria, fungi, and algae. The ability to be decomposed by microorganisms depends on the chemical structure rather than the origin: petroleum-derived plastics can also be biodegradable [28]. A compostable plastic is a plastic that undergoes degradation through biological processes during composting to yield CO_2_, H_2_O, inorganic compounds, and biomass at a rate that is consistent with other compostable materials, and leave no visible, distinguishable or toxic residue [29]. Composting is a biological process in which the organic material is decomposed primarily by microorganisms, to produce a soil-like substance, called humus. The composting conditions are affected by environmental factors, such as water or moisture content, temperature, acidity, enzyme specificity; or polymer factors, such as polymer structure and chain flexibility, crystallinity, and molecular weight, as well as copolymer compositions, size, and shape [30].

**Figure 2:**
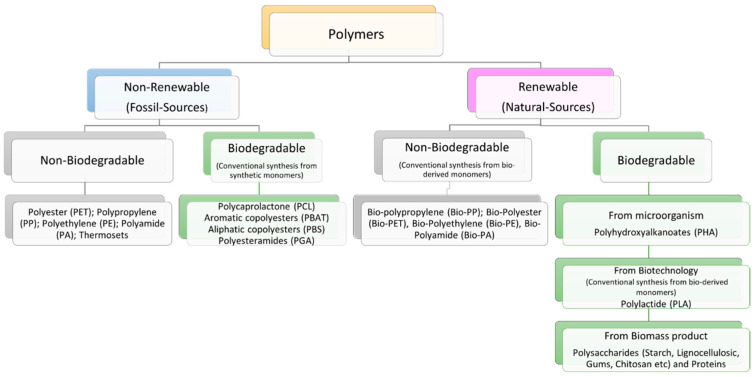
Schematization of biodegradable and non-biodegradable plastics [31,32].

Plastics can be broadly classified into the four categories listed below (Figure 2): (i) fossil-based and non-biodegradable plastics (PET, PP; PE; PA, thermosets); (ii) fossil-based and biodegradable (PCL, PBAT), (iii) natural-based and non-biodegradable (bio-PP, bio-PE, bio-PET), and (iv) natural-based and biodegradable (PLA, PHA, lignocellulose, starch, proteins) [31,32].

**Figure 3:**
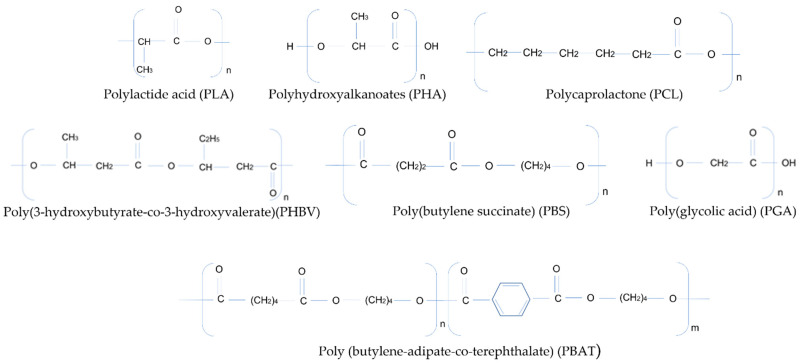
Chemical structure of main biodegradable polymers.

A transition to more sustainable plastics requires the development of systems not derived from fossil resources and towards carbon-neutral blocks, with specific functional and recyclable properties. In general, there are three methods to synthesize biopolymers from renewable sources: to make use of natural polymers that can be modified but remain similar to a large extent (such as starch), to produce monomers from fermentation that follow polymerization, and to produce biopolymers directly through microorganisms [33]. The following biodegradable products are currently on the market (Figure 3):

- Poly(lactic acid) (PLA) is one of the most commercially successful bio-plastics. The monomer (lactide acid), is produced through fermentation from renewable sources such as starch or sugar. Other carbohydrate resources include: leaves, stems and stalks from corn fiber, corn stover, sugarcane bagasse, rice hulls, woody crops, and forest residues [34]. Jem and Tan (2020) estimated the global production volume of PLA in 2019 to be around 190,000 tons [20]. However, their modest thermal, mechanical and rheological characteristics, as well as their incompatibility with the process and recycling technologies [35], limit the practical application of PLAs, making necessary their copolymerization or blending with other polymers;
- Polyhydroxyalkanoates (PHA) accumulate inside the cells of microorganisms as granules for energy storage under restrictive nutrient conditions and high concentrations of carbon [28];
- Poly(3-hydroxybutyrate-co-3-hydroxyvalerate)(PHBV) is obtained by the biosynthesis of diverse bacteria in the presence of specific nutrients (e.g., nitrogen, phosphorous, or sulfur) and an abundance of carbon sources [36];
- Polycaprolactone (PCL) is obtained through the condensation of 6-hydroxycaproic (6-hydroxyhexanoic) acid or ring-opening polymerization (ROP) of ε-caprolactone (ε-CL). Industrially ε-CL is produced from the oxidation of cyclohexanone. The products ε-CL and 6-hydroxyhexanoic acid are also intermediary products in the oxidation process using microorganisms [37];
- Poly(butylene-adipate-co-terephthalate) (PBAT) is produced through the polycondensation reaction of butanediol (BDO), adipic acid (AA), and terephthalic acid (PTA), using general polyester manufacturing technology [38];
- Poly(butylene succinate) (PBS) is produced through the polycondensation reaction of succinic acid (or dimethyl succinate) and butanediol (BDO). The monomers can be derived from fossil-based or renewable resources. Succinic acid can be obtained through fermentation of the microorganism; renewable feedstocks, such as glucose, starch, or xylose; or by chemical process via the hydrogenation of maleic anhydride [39];
- Poly(glycolic acid) (PGA) is attained by the direct poly-condensation polymerization of glycolic acid, via the solid-state polycondensation of halogen acetates, by reacting formaldehyde with carbon monoxide, or via the ring opening polymerization of glycolide, the cyclic dimer of glycolic acid (current industrial route) [40];
- Biomass: polysaccharides (starch, lignocellulose, chitosan, gums) and proteins (silk, collagen, soy, zein).

In recent years, there has been a growing trend towards the replacement of fossil-based conventional plastics with identical molecules derived from renewable sources [41]:

- Bio-poly(ethylene) (bio-PE) is produced from the dehydration of ethanol from sugar to produce ethylene, which, upon purification and polymerization. produces polyethylene [42];
- Bio-polypropylene (bio-PP). Biomass can be converted into bio-based propylene by multiple processes, such as cracking, gasification, fermentation, metathesis, and dehydrogenation. Methanol, isopropanol, ethanol, butanol, and glycerin, are good candidates for propylene synthesis [43];
- Bio-poly(ethylene terephthalate) (bio-PET) is obtained through bio-based ethylene glycol as the monomer. Its precursors, terephthalic acid (TA) and ethylene glycol (EG), are both produced from biomass (forest residues, corn stover). Bis(hydroxyethyl)terephthalate (BHET) is synthesized from ethylene glycol (EG) via a transesterification reaction with dimethyl terephthalate (DMT) or an esterification reaction with terephthalic acid (TA). Next, BHET pre-polymerization and melt polycondensation form low-Mw PET or solid-state polymerization to produce high-Mw PET [44];
- Bio-poly(trimethylene terephthalate) (bio-PTT) is produced from biobased 1,3-propanediol (1,3-PDO) [41];
- Bio-polyamide (bio-PA). The main source of bio-based monomers (11-aminoundecanoic acid, 1,8-octanedicarboxylic acid, 1,10-decanediamine) is castor oil derived from oil crops. Other biomass (sugar, starch, lignocellulose)-derived monomers are adipic acid, caprolactam, and 1,4-butanediamine [45].

Therefore, in light of sustainable green manufacturing, future developing fabrics should be biodegradable. Fabrics made with similar fiber characteristics, but derived from renewable resources, such as bio-PET and bio-PA, could be used as alternatives to current fabrics made with synthetic fibers. However, the debate over the potential pollution induced during the latter’s life cycle remains open, as the latter, not being biodegradable, can equally cause an aggravation of problems already highlighted with the use of fossil-based filaments during the life cycle and at the end of their use [46]. 

Nevertheless, the step forward in reducing the environmental impact during the production of fibers from renewable sources cannot be neglected. The lack of biodegradability could not be considered an issue as durable bio-plastics have identical performance compared to petroleum-based ones, and could be directly applied in existing recycling systems. Furthermore, during the production of durable bio-plastics, although these are still more expensive, a large reduction in greenhouse gas (GHG) imprint emission is obtained compared to petro-equivalents [47]. Life-cycle assessments (LCAs) for bio-PE and PLA bioplastics were compared to those of two fossil-derived plastics, high-density polyethylene (HDPE) and low-density polyethylene (LDPE), in terms of greenhouse emissions (GHG) and fossil fuel consumption (FFC). The results demonstrated the benefits of bio-based plastic pathways over fossil-based pathways by showing GHG equal to −1.0 and 1.7 kg CO_2_e per kg for bio-PE and PLA with no biodegradation, compared with 2.6 and 2.9 kg CO_2_e per kg for LDPE and HDPE; and FFC equal to 29 and 46 MJ per kg of bio-PE and PLA, compared with 73 and 79 MJ per kg of LDPE and HDPE. However, despite the benefits of biogenic carbon uptake, at the end-of-life, PLA emissions were increased from 16% to 163%, passing from composting to landfill because less CH_4_ was emitted in the composting gas [48].

A comparison between the properties of bio-based and traditional plastics is presented in Table 1.

## 4. Traditional Textile Materials

One of the most basic human needs since the Prehistoric Age has been to cover and protect the body from cold and/or bad weather. Many anthropological and archeological studies have demonstrated the practical and symbolic importance of fabrics throughout human history, particularly in rituals, events, and ceremonies [62]. The first woven fibers were discovered in Egypt during the Neolithic era: they were made of very fine-quality hemp, i.e., linen, in a complex pattern (“brocaded”) with fringes at the edges [63]. There is evidence of a wool trade in Iran, and there were woolen garments in Babylon. Around 3000 BC, Egypt and India had well-developed spinning and weaving of linen and cotton. During the Bronze Age (2000 BC), discoveries of animal fibers for burial were made in Northern Europe. Turkey became skilled in carpet manufacturing during the Middle Ages; Palermo (Sicily) became famous for the production of elaborate silk and gold fabrics, and Lyon (France) became the most important silk manufacturing center in Europe. Prior to the industrial revolution, the textile industry grew more as a trade in fine ornamental yarns and beautiful textiles than as a result of technological advancement. However, W. Lee of Woodborough invented the knitting machine in England at the end of the 1500s, followed by cotton gin in 1793, the flying shuttle for weaving, invented by John Kay, and the mechanized loom, invented by Edmund Cartwright [64]. Textile production, based on both natural and synthetic fibers, has grown significantly in tandem with population growth, as well as with the economic and commercial aspects of fashion and style. 

Textile fibers can be divided into two main categories: natural and synthetic fibers [65]. The former originate from animals, vegetables/plants, or mineral sources. Examples of animal-based fibers are wool, silk, and hair (alpaca, goat, horse), whereas plant-based fibers are constituted by seed, bast, leaf, wood; finally, mineral fibers include asbestos, fibrous brucite, and wollastonite [66]. As confirmed in a recent study by Uddin [67], natural fibers better meet human requirements in view of comfort and aesthetics. Cotton, silk, and wool are the three most common natural textile fibers used in clothing production. Recently, the market has witnessed the introduction of “organic” cotton, which is cotton grown without the use of chemicals, pesticides, or fertilizers. This has shifted production toward more sustainable development. Organic cotton is primarily produced in countries such as Turkey, China, India, and United States [68]. Wool fiber is well known for its warmth and is commonly used in winter clothing, with renewability and recyclability characteristics that contribute to its popularity in this industry. Silk fiber is well-known for its unique softness and low linear density, despite being produced in much smaller quantities than cotton and wool.

Synthetic fibers, also known as man-made fibers, are typically made from synthetic materials, such as petrochemicals (polyamide, polyester, polypropylene) [65]. However, some synthetic fibers, such as rayon, modal, and the more recently developed Lyocell (a regenerated cellulose fiber made from dissolving pulp), are made from natural cellulose (bleached wood pulp). Synthetic fibers can often be produced at a lower cost and in greater quantities than natural fibers. For clothing, natural fibers can provide some advantages, such as comfort, over their man-made counterparts [69].

**Figure 4:**
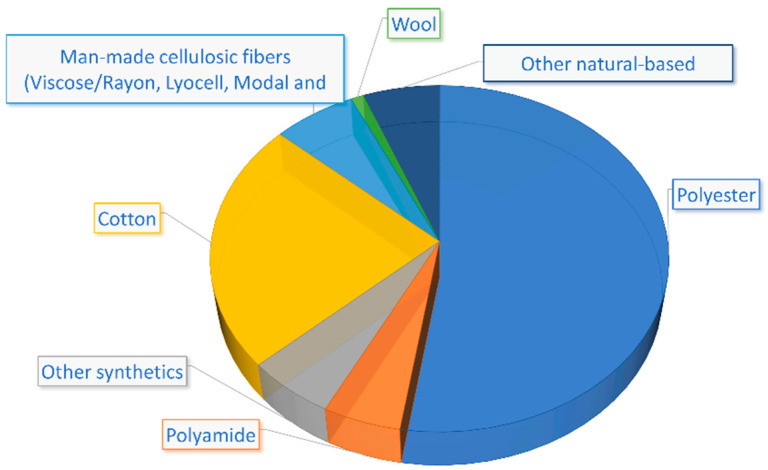
Global fiber production in 2019 (as reported in [70]).

In 2019, more than 60% of the world consumption of natural and man-made fibers belonged to synthetic fibers, primarily made from polyester, followed by cotton, cellulosic, and wool [70]. Data on global fiber production are presented in Figure 4 [70]. In 2019, China was the main consumer of natural and synthetic fibers, employing more than half of the total, followed by other Asian, North American, and European countries [71]. 

## 5. Polluting Aspects by Textile Industry

The manufacturing process of textiles consists of four primary stages: yarn production, fabric production, textile production, and finishing treatment. 

Pesticides, insecticides, and fertilizers are used in raw material treatments for cellulose- and protein-based natural fibers. After the first step, which is the removal of impurities, a series of continuous operations is followed. This series consists of blending, mixing, cleaning, carding, drawing, roving, and spinning; these processes are mainly mechanical, not requiring chemical applications [67]. By contrast, monomers, chemical agents, precursors, catalysts, and a variety of auxiliary chemicals are used in the preparation of synthetic polymer-based yarns [67]. 

Three general stages can be distinguished in man-made manufacturing yarn processes: the preparation of the spinning fluid from solid polymers or monomers; fiber spinning by extrusion; and, subsequently, solidification, mechanical, thermal and chemical treatment to improve properties [72].

Several processes are applied during the production of fabrics, including: sizing chemicals to improve absorbent capacity; oxidizing agents to improve strength, hygroscopicity, dye absorbency, and brightness; the use of pigment and chromophore agents, as well as dyes, to improve color [8]. 

**Figure 5:**
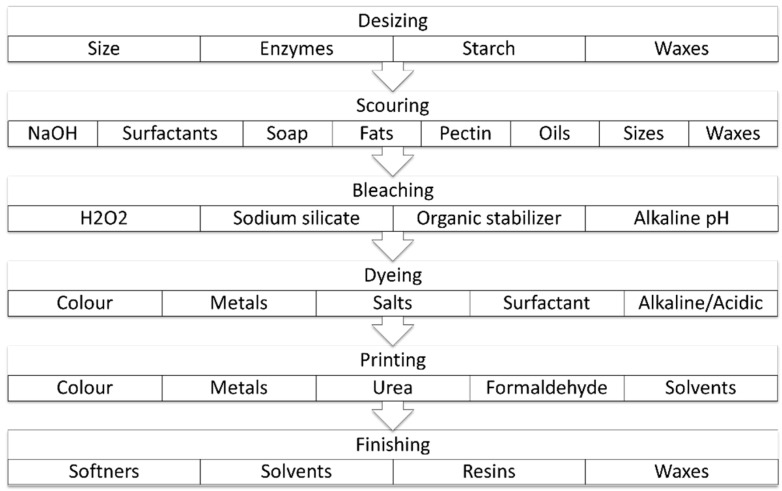
Schematization of main pollutants employed in stages of textile wet processing. Reproduced from [75]. Copyright (2016), with permission from Elsevier Ltd.

The ultimate appearance and aesthetic properties of textile materials are determined by textile finishing, which has the potential to change various physical and chemical properties of textile materials in response to consumer demands [73]. Specific finishing treatments have been used to impart water and oil repellency, antibacterial resistance, and flame resistance [8]. Textile finishing, including washing, bleaching, dyeing, and coating, is applied to bulk textiles or garments after weaving and/or synthetic material production [74]. These processes are conventionally performed through typical pad-dry-cure techniques, consuming intensive amounts of energy and massive quantities of water, usually discharged as effluent [74]. The pollutants involved in each operation of the textile industry are summarized in Figure 5 [75].

With the growth of the world’s population and rising living standards, large amounts of production and post-consumer fiber waste have accumulated in nature or in landfill [76]. Fibers, yarns, fabric scraps, and apparel cuttings are examples of production waste generated by fiber producers, textile mills, and fabric and apparel manufacturers. Pre-consumer waste are products with mistakes in design, faults, or incorrect colors, while post-consumer waste refers to garments or household items that the owner no longer requires and chooses to discard [8]. 

The cleaning of clothing made of synthetic fabrics appears to be a significant source of contamination for sewage due to the production of micro- and nano-sized plastic fragments released in washing water. Recently, microplastics have been revealed as new sources of pollution in the oceans. Because microplastics are not visible to human eye, and do not degrade in an aqueous environment, they can become food for plankton and other organisms that live in the oceans by entering the food chain [77].

## 6. Properties of Bio-Based Fibers and Fabrics

Choosing an appropriate fiber among various renewable fibers derived from biomass necessitates an awareness of sustainability based on the product’s GHG imprint and the energy requirements of its production and distribution [46]. The selection of material should be based on the product’s value chain, previous use in clothing, and a comparison of its physical parameters with the most commonly used synthetic fibers derived from PET (terylene, dacron, etc.) [46].

### 6.1. Biodegradability

**Figure 6:**
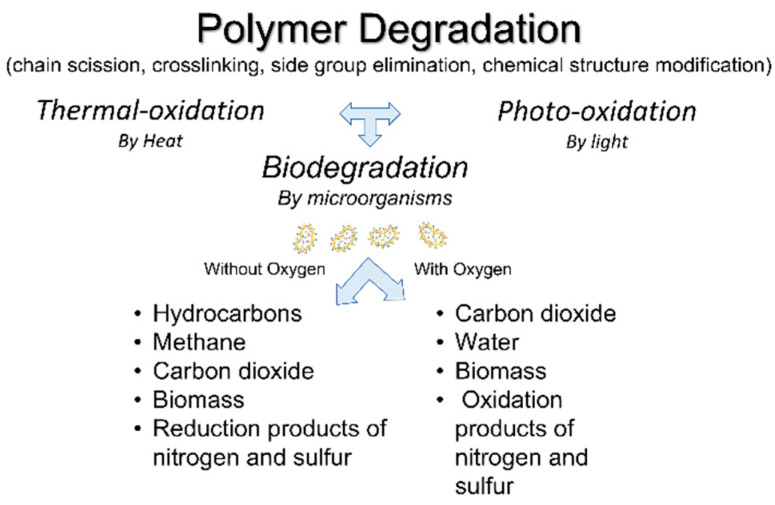
Products of polymer biodegradation under aerobic or anaerobic conditions.

Polymer degradation occurs as an effect of different mechanisms (photodegradation, thermo-oxidative degradation, and biodegradation) throughout chemical reactions such as chain scission, crosslinking, side group elimination, chemical structure modification [78]. Biodegradation consists in the breakage of organic matter by means of microorganisms through two main pathways developed under aerobic (in the presence of oxygen) and anaerobic (in the absence of oxygen) conditions [79] (Figure 6). The final products of aerobic biodegradation are carbon dioxide, water, biomass, and oxidation products of nitrogen and sulfur; whereas, in the case of anaerobic biodegradation, hydrocarbons, methane, carbon dioxide, biomass, and reduction products of nitrogen and sulfur are released [80]. 

Environmental biodegradation occurs in the case of uncontrolled conditions (temperature, moisture, pH, nutrients, oxygen level, presence of organisms, and composition of the waste), without human involvement. If conditions are inadequate, the biodegradation process leads to waste accumulation [79]. 

Once discarded in the environment, polylactide is hydrolyzed into low-molecular-weight oligomers, and then converted into CO_2_ and H_2_O by microorganisms present in the environment [81]. However, microorganisms able to degrade PLA are not widespread in the soil, making PLA biodegradation less feasible than that of other polyesters, such as PHB, PCL, and PBS [82]. 

Aliphatic bio-polyesters (PLA; PGA, PCL, etc.) have been commonly used in biodegradable products. The hydrolytic and/or enzymatic chain cleavage of these materials leads to αhydroxyacids, which could be assimilated by the human body or in composts [83]. However, poor mechanical properties and degradation time restrict the applications of these biopolymers. Copolymerization or blending have been designed to increase the performance enabling a range of mechanical properties and degradation rates [83]. The fibers of PLA-PCL, PGA-PCL, PDO (polydioxone) and PGA, with two different diameters (150 µm and 400 µm), were characterized in terms of degradation rate under three different environments (water, NaCl and PBS). After immersion in an aqueous medium, the first phenomenon to occur was the penetration of water from the surface to the center due to the negative gradient of water concentration (pure diffusion), which was faster compared with degradation. Therefore, it was considered that the hydrolysis of ester bonds started homogeneously. Despite the usual low degradation of PCL, PGA-PCL, the corresponding fibers were the fastest in terms of weight loss, followed by PGA, PDO, and the less biodegradable PLA-PCL [83].

Cotton fibers are mostly made of cellulose, which is biodegraded by microorganisms that secrete enzymes called cellulases. Cellulases catalyze the hydrolysis and oxidation of the cellulose molecular chain into cellobiose units, which then degrade into glucose and glucose derivatives, both of which are non-hazardous to the environment [84]. The biodegradation of cotton fabric is affected by textile finishing (silicone softener, durable press, water repellent, and a blue reactive dye). The rate of degradability of textile microfibers during laundering decreased with durable press- and water-repellant finishing treatment. The presence of crosslinking (durable press) and hydrophobicity (water repellent) on the surface slows down the initial adsorption of enzymes excreted by microorganisms in the inoculum. Therefore, the use of manufacturing techniques such as derivatization, blending, and coating to improve bio-based material performance in practical applications affects the environmental impact of the final product [85].

Experimental evidence of the aquatic biodegradation of cotton, rayon, and polyester-based fabrics has demonstrated that during, laundering cellulose-based fabrics releases more microfibers (0.2–4 mg/g fabric) than synthetic textiles (0.1–1 mg/g fabric). However, cotton and rayon fibers degraded in aquatic conditions faster than polyester fibers, which, by contrast, persisted in the environment for a long time [86].

The biodegradation of wool (natural keratin fiber), cotton (a natural cellulose fiber), and fiber of PLA was evaluated at 35 °C for 42 days to determine the time-dependent changes in weight loss, strength loss, and morphology under natural soil and aqueous medium conditions. The results made it possible to determine that the degradation rates in natural soil were higher than in aqueous medium, listed in the following order: cotton > wool > PLA fiber. This led to the conclusion that natural fibers degrade more easily than man-made biodegradable PLA fibers [87]. 

Based on a study by Egan and Salmon [79], the percentage biodegradation of textile fibers in various environments can be summarized as follows: (i) cotton fibers are biodegradable in soil (180 days) and anerobic conditions (30 days), and semi-biodegradable in compost (45 days); (ii) PLA-based fibers are biodegradable in compost (45 days), semi-biodegradable under anerobic conditions (30 days) and seawater (90 days), and completely non-biodegradable in soil (180 days); (iii) PET fibers are never biodegradable.

### 6.2. Mechanical Performance

Polylactide acid (PLA) is one of the most promising biopolymers in textiles, since it possesses similar characteristics to synthetic fibers, with superior biodegradability compared to other biopolymers. It is soft to the touch, features a silky sheen, and offers good durability. However, the breaking strength of pure PLA was very low, making it necessary to set specific parameters for the production and processing of PLA fibers [88], or for blending it with other polymers [89]. 

Persoon et al. [90] analyzed the effect of draw ratio and temperature on the tensile and thermal properties of melt-spun monofilament and multifilament fibers from PLA. The fibers were produced by melt spinning and subsequent solid-state, by choosing two matrices, different in viscosity and molecular weight. The final results demonstrated that the melt draw ratio, solid-state draw ratio, and drawing temperature strongly affected the mechanical properties of PLA fibers. By increasing the solid-state ratio, the orientation and crystallinity were increased. The authors concluded that a higher solid-state draw ratio was preferable when manufacturing PLA fibers with higher orientation, crystallinity, and tenacity, and that as a result, the melt draw ratio should be reduced accordingly.

Weft-knitted single-jersey fabrics were prepared by combining 100% PLA yarn or PLA/lyocell blended yarns. By increasing the proportion of the lyocell fibers, the bursting strength of corresponding fabrics was increased (bursting strength of basic PLA of 300 kPa), although it remained lower than that of PET/cotton blended fabric [91].

Endowed with strong antimicrobial activity and excellent biocompatible and biodegradable properties, PHBV can be represented as a potential candidate for replacing petroleum-derived polymers. Unfortunately, the mechanical strength, water sorption and diffusion, and electrical and/or thermal properties are all lacking, necessitating its use in conjunction with other polymers [92]. In this regard, PLA/PHBV blends (100% bio-based and fully degradable) have been investigated in terms of spinnability, as well as their mechanical and thermal characteristics. The results made it possible to demonstrate the promising application of these fibers to textiles: the addition of PHBV was shown to be compatible with PLA and increased the flexibility of the blend. The highest tenacity and shrinkage were recorded with PLA/PHBV blends of 95/5 and 90/10. At these concentrations, the fibers were subjected to knitting to prepare knitting socks [93]. A further investigation of dyeing processes of textiles made from PLA/PHBV was also performed by determining the effects of different dyes, dyeing temperatures, dyeing times, pH and liquor ratio on the dye exhaustion in the dyeing process, color fastness, and mechanical properties. The final results confirmed commercially acceptable levels of the measured characteristics, and the feasibility of the designed dyeing process. The calculated amount of energy required for the whole dyeing was comparable with that of polyethylene terephthalate [94]. Other studies went further in testing the antibacterial ingredient, polyhydroxy butyrate (PHB), of the PHBV material as a potential candidate for antimicrobial activity, in textile fabrics made from poly (hydroxybutyrate-co-hydroxy valerate)/polylactide acid (PHBV/PLA) with natural cotton fibers [95]. The effects of blend yarn, fabric structures, and distributions of fibers on the antimicrobial properties of the knitted fabrics were analyzed.

A comparison of the mechanical, thermal, and surface properties, as well as the anti-bacterial behavior, of five different types of synthetic multi-filament yarn and the corresponding knitted fabrics was presented in the work of Huang et al. [96]. Three bio-based materials (PLA/PHBV, PLA, Cupro) and two petroleum-based materials (PET, PA6) were considered. The final results made it possible to attest that the bursting strength, extension, and recovery of jersey-knitted fabrics from PLA/PHBV yarns satisfy industrial requirements. For these fabrics, a bursting strength of 375.8 kPa was measured, which was higher than that of PLA fabrics, similar to that of PA 6, and significantly lower than PET fabrics. Dyed PLA/PHBV knitted fabrics displayed the worst abrasion resistance (2250 rubs) compared with other fabric samples (the highest: PET fabrics with abrasion resistance > 50,000 rubs), promising tearing property, and good air permeability, as well as excellent antibacterial performance against staphylococcus aureus, klebsiella pneumoniae, and candida albicans.

### 6.3. Breathability and Comfort

“Clothing is comfortable when we are unaware of it” [97]. Factors that increase the awareness of wearing a garment, such as its pressure on the skin, its temperature, and its wetness, decrease the comfort [98]. Depending on personal choices, discomfort arises when the pressure approaches that of diastolic blood pressure (80 mmHg), three-quarters of the body surface is covered by liquid sweat, and a constant core temperature is maintained at different work rates for the body [97]. This means that during human activity, the generated heat should be transferred to the environment. The main mechanism of heat transport, especially during physical activities, is water evaporation and its transport to the surroundings [98].

Polylactide acid has a higher natural hydrophilicity than most other thermoplastic polymers, including polypropylene, nylon, and PET, because water molecules can enter in PLA macromolecules through polar oxygen linkages [99]. Although PLA fibers are not as wettable as cotton, the improved wettability characteristics of these fibers determine a larger moisture vapor transmission compared to PET or nylon fiber-based fabrics. This aspect allows the greater “breathability” of garments such as shirts, dresses, underwear, and shoes composed of PLA-based fibers when these fibers are used in place of fibers such as PET or nylon [99].

### 6.4. Antibacterial Properties

Resistance against microbial attack is one of the important aspects of the textile industry. A good contact area and the absorption of moisture are the two main causes of microbial growth, which leads to unpleasant odors, dermal infections, allergic reactions, and fabric deterioration. Thus, the incorporation of antimicrobial agents on textile products capable of overcoming these issues is critical [100]. Chitin and chitosan are highly versatile biomaterials that have gained widespread attention due to their unique properties, such as nontoxicity, biocompatibility, biodegradability, low allergenicity, biological activity, low cost, and so on [101]. One of the most common applications of chitosan in the textile industry is as an antimicrobial agent, given its ability to provide protection against allergies and infectious diseases, as well as moisture retention and wound-healing capabilities [102]. Because of the presence of reactive amino and hydroxyl groups along the backbone, chitosan has some intriguing properties for use in textile dyeing and finishing. However, low water solubility at neutral pH and poor durability on textile surfaces limit the widespread use of chitosan [103]. Experiments have been conducted on the use of crosslinking and graft polymerization to chemically modify chitosan to produce water-soluble bioactive derivatives. For example, glutaric dialdehyde was chosen as the crosslinking agent to chemically bond chitosan to cotton fabric by verifying the antibacterial behavior of chitosan against Escherichia coli and the Hay bacillus. It was found that 0.3 g/L chitosan solution effectively inhibited W. Escherichia coli, whereas 0.5 g/L chitosan solution was required against Hay bacillus [104]. 

The worldwide epidemic caused by the coronavirus 2 (SARS-CoV-2) pandemic has focused attention on rules, attitudes, and best practices for virus disease prevention. As a result, there has been a surge of interest in the development of surfaces that inhibit or prevent the adhesion of these microorganisms [105]. The health risks posed by the SARS-CoV-2 viruses and the need to protect the environment have shifted the focus to safe and durable antiviral and antibacterial textiles made from bio-based, renewable materials. New fibers and environmentally friendly advanced technologies to improve the mechanical properties and the antimicrobial behavior of textiles have been examined in [105]. Chemical treatment to modify natural and synthetic fibers is one of the viable and promising methods used in textile coating and finishing to improve their antibacterial, ultraviolet-protection, flame-retardant, and anti-static properties [106]. However, the involvement of hazard chemicals during chemical modification treatments raised environmental concerns by encouraging the use of a variety of natural and biodegradable coatings for wet chemical surface modification [107]. A series of novel chitosan-based water-dispersible polyurethanes have been experimentally assessed for upgrading the antibacterial activity of polyester/cotton textiles [108]. Nano-chitosan-polyurethane dispersions (NCS-PUs) were prepared via polymerization processes and applied on polyester cotton fabric (PCF) as a finishing agent, using the pad dry cure technique. Antibacterial activity, ultraviolet protection factor, tear, and tensile strength were measured for both dyed and printed textiles, demonstrating the NCS-PUs treatment’s usefulness and effectiveness in increasing the desired properties of coated samples. The gradual increase in concentration of chitosan into the PU backbone significantly enhanced resistance against *B. subtilus*, *S. aureus* (gram-positive) and *E. coli* (gram-negative).

### 6.5. Flammability

A comparison of the flammability properties of PLA and PET fibers showed low flammability and less smoke generation than PET [109]. However, the high flammability of PLA is well known, which can result in the production of toxic gases during combustion in vitiated atmospheres [110]. As a result, PLA’s flame-retardant properties have become an issue, limiting the scope of its applications [111]. Although most systems have not yet been commercialized, phosphorus-based PLA formulations have proven to be quite effective at increasing the flame-retardant features of corresponding PLA-based materials [110]. In this regard, intumescent formulations mainly composed of acid and carbon sources was analyzed in the work of Cayla [112]. Lignin from wood waste was selected as the carbon source, and added in different PLA-based preparations, to ammonium polyphosphate (AP) by melt extrusion, and then hot-pressed into sheets. The spinnability, thermal behavior, and flame retardancy of the developed PLA formulations containing lignin and/or ammonium polyphosphate were explored. MFI results confirmed that not all the formulations were spinnable and endowed with an increase in thermal flame-retardant properties. Depending on the concentration, the presence of ammonium polyphosphate in blends may not only degrade the macromolecular PLA chains, but also change interactions between all of the compounds. This may result in an increase in free volume of the PLA matrix and lignin agglomeration. A type of regenerated cellulose fiber that is widely used as bio-derived material in textiles, public transportation, electrical equipment, and building industries is viscose fibers. It offers numerous advantages, such as biodegradability, excellent air permeability, non-toxicity, low cost, and environmental advantage, with the main disadvantage being its high susceptibility to ignition. In order to overcame this drawback, alginate fibers were added to viscose yarns, and the effect of the content on the thermal stability, flame retardancy, and combustion behaviors of the prepared viscose/alginate-blended nonwoven fabrics, were investigated. The final results confirmed changes in the thermal stability of developed nonwoven fabrics by decreasing flammable gas emissions, thus improving the flame retardancy and inhibiting the smoke release [113].

The tenancy values of common natural, man-made, and bio-based fibers, are summarized in Table 2. The melting point and indications of moisture absorption in terms of moisture regain (the weight percentage of water in a material versus the material’s dry weight) or overall moisture management capacity (an index between 0–1 expressing the ability of the fabric to manage the transport of liquid moisture, with 0–0.2 being poor, 0.2–0.4 being fair, 0.4–0.6 being good, 0.6–0.8 being very good, and 0.8–1 being excellent) are also shown.

## 7. Recent Applications of Biopolymers to Textiles

### 7.1. Textile Finishing and Treatment

Conventional textile wet processing poses a significant challenge to the development of eco-friendly processes and green products given the negative effects on human health and the environment due to the requirement of a significant amount of energy for heating, drying, and/or steaming, in addition to the machinery, resulting in an increase in greenhouse gas emissions and carbon footprint [120]. Recently, there has been an increasing awareness of hygienic lifestyle, concern over carbon and water footprint, and a desire to meet consumer demands at the same time, ensuring the sustainability of our eco-systems. The application of green chemistry principles and aspects of cleaner production have been considered for preserving the environment, economy, and society [121]. The use of enzymes in the textile pre-treatment of natural fibers, such as depilling, desizing, scouring, and so on, has already proven to be a highly profitable and sustainable alternative to the harsh toxic chemicals used in the textile industry [122]. Enzymes are proteins made up of amino acids, responsible for thousands of metabolic processes that sustain life. They act as catalysts, accelerating chemical reactions in highly specific and efficient ways while not altering or being consumed [121]. Different enzymes are already used in experiments for the textile industry, and they are able to impart specific functional features to treated textiles: chitosan [103], cyclodextrin [123], alginate [124], or plant-based bioactive materials (aloe vera) [125], essential oils (jasmine) [126], natural dyes extracted from different parts of plants such as bark, leaf, root, and flowers containing common coloring materials such as tannin, flavonoids, and quinonoids [127].

In order to impart shrink resistance to wool fabric, an eco-friendly treatment involving a sequential combination of enzymes followed by polysaccharide-based bio-polymers was proposed by Kadam et al. [128]. For the experiments, wool fabric was considered and treated firstly with laccase and protease enzymes, and then coated with three polysaccharide biopolymers (chitosan, wheat starch, and gum arabic) using the pad-dry-cure technique. On the developed samples, a characterization based on microscopy and IR spectroscopy, tensile, frictional, and bending analysis was performed. Except for friction, shrink-resistance treatment has no effect on tensile or bending properties. The combination of treatments preserved the whiteness of the wool fabric while reducing its yellowness. The result obtained in the presence of chitosan treatment was found to reduce the wool fabric shrinkage to <4%, which is deemed comparable to traditional chlorine-based methods.

UV-B radiation (wavelengths from 280 to 315 nm) results in harm to humans, particularly in terms of its effects on the eyes and skin [129]. It can cause damage to DNA and RNA structures in humans, leading to helix distortion and alterations in transcriptional programs [130]. In this regard, Dominguez-Pacheco et al. [131] examined the effect of adding natural pigments, extracted from the cooking of commercial white corn kernels, on the improvement of fabric protection against UV radiation. The optical absorption spectra of natural pigments and treated textiles were obtained using photoacoustic spectroscopy. During the cooking of agricultural grains, several components, such as phenols and flavonoids, contained in the superficial layers were released. Maceration and microwave oven-assisted extraction were the two adopted methods to capture the natural pigments from the samples. The experimental results confirmed the higher optical absorption coefficient of textiles treated with bio-based additives, compared to textiles to which a chemical anti-UV agent had been added, and a wider band in the optical penetration length. This indicated that the previous systems possessed greater opacity and less penetration by UV light.

### 7.2. Printing on Textiles by Fused Deposition Modeling (FDM)

A recent development in 3D printing processes increased the potential of this technology by promoting its accessibility and providing a new platform for design, customization, and innovation [132]. Many fashion designers are taking advantage of this innovation by producing textiles, clothing, jewelry, notions, or shoes [133]. Sophistication and fidelity to style have been added to the printed textiles [134], but functional characteristics such as rigidity [135] and abrasion resistance [136] have also been imparted. In the work of Singh et al. [137], cotton-knitted fabric and tulle net fabric based on nylon were selected for better deposition of the fused plastic material inside the fabric. The effect of the infill percentage on the adhesion property was investigated. The authors concluded that the fiber direction in the fabric and the first layer played a larger role in affecting the adhesion properties than the platform temperature.

To improve the environmental footprint of the production process for smart and functional textiles by avoiding unnecessary use of water, energy, and chemicals while minimizing waste, Hashemi Sanatgar et al. [138] proposed 3D printing as a more cost-effective textile functionalization process than conventional printing processes such as screen and inkjet printing. They investigated the adhesion characteristics, depending on the fabric and filler type, of polymers and nanocomposite layers, printed directly onto the textile fabrics by using the FDM method. Nylon was printed on polyamide 66 (PA66) fabrics, polylactic acid on PA66 and PLA fabrics, and finally nanocomposites of PLA and carbon black or multi-wall carbon nanotubes on PLA fabric. The results demonstrated that different 3D printing process variables, such as the extruder temperature, platform temperature, and printing speed could all have a significant impact on the adhesion force. The breaking strength of 3D printed layers was reduced by increasing the extruder temperature. This was interpreted as a sign of increased brittleness caused by higher processing temperatures. Next, as the printing speed increased, the adhesive force decreased. In fact, improving the printing speed reduced macromolecule penetration into the fabric, resulting in cohesive forces, which are greater than adhesive ones. No significant linear effect on the adhesion force of the platform temperature (chosen close to the glass transition temperature) was verified. This was attributed to the poor mobility of macromolecules, which reduced the diffusion of the printed polymer in the fabric structure by leading to negligible adhesion. The platform temperature was found to play an important role in increasing the adhesion when the value was higher than the glass point. The highest value of the adhesive force was verified for samples made from PLA-based nanocomposites applied to PLA fabrics.

**Figure 7:**
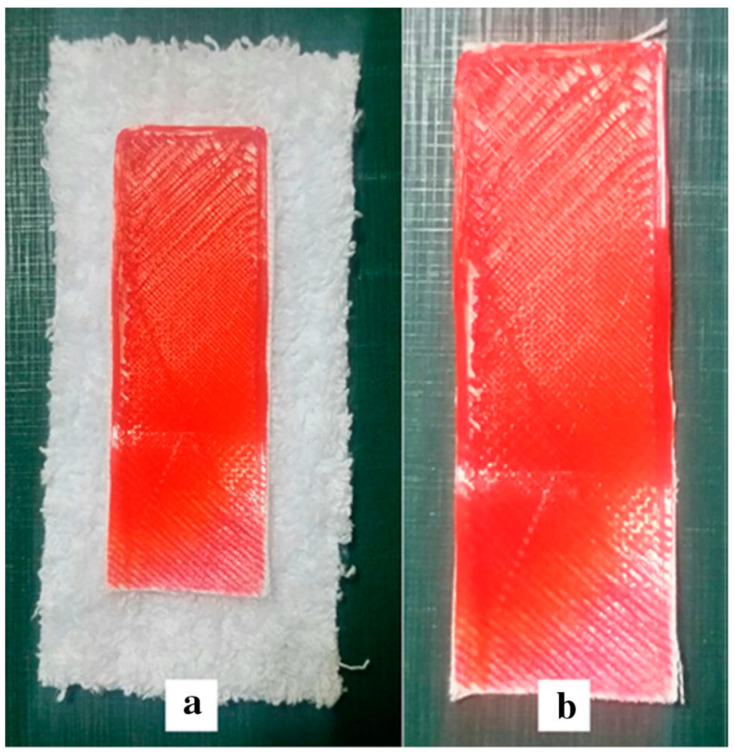
Example of PLA printed on cotton fabric (**a**) before cutting (**b**) after cutting the edges. Reprinted [139].

By contrast, Mpofu et al. [139] concentrated their study on fabric properties affecting the adhesion of 3D printed PLA polymer onto selected fabrics (acrylic, cotton, polyester/cotton blend and polyester) (Figure 7). The effect of fabric areal density, warp and weft count, fabric thickness and roughness, ends/inch and picks/inch, was analyzed through a regression model. The former four parameters were discovered to have a positive effect on the investigated property, while the last were revealed to exert a negative effect. In terms of fiber types, acrylic-based fabrics had the highest adhesion force to PLA, while polyester-based fabrics had the lowest.

Many clothing companies have begun to use 3D printing to create accessories and soles. However, due to a lack of flexible materials, it is still not possible to produce ready-to-wear clothing. In this regard, Uysal and Stubbs [140] developed a new method for printing textile-like surfaces. Different flexible structures were made of layers by combining different materials, PLA and lay-foom (made from a rubber-elastomeric polymer and a PVA-component), numbers of layers, and repeating patterns (polygon, rectangles, floral). Sewing patterns were printed and assembled into a three-dimensional garment using a single printing step instead of typical production steps in the textile supply chain, such as fabric production, dyeing, colour printing, cutting, and the application of other components, such as inner linings.

### 7.3. Treatment of Dye-Contaminant Water

One of the aspects of textile production that causes the most pollution is the contamination of fresh water, particularly from the dye treatment [141]. This reduces the quality of water and renders it unfit for consumption by inhabitants. Some dyes are more resistant and difficult to degrade completely by using photolysis, biological and chemical decomposition, and other ordinary approaches [142]. Advanced treatment technologies, such as adsorption processes, advanced oxidation processes (AOPs), and membrane processes have been shown to be promising alternatives for micropollutant removal; however, high operating costs and the formation of by-products and concentrated residues limit their application [143]. From an environmental point of view, it is also essential to develop new technologies for the wastewater treatment and recycling of dye-contaminated water. A recent review by Sirajudheen et al. [144] analyzed the applications of chitin and chitosan to the adsorption of textile dyes from water. Chitin and chitosan are among the most abundant natural biopolymers on the planet. They are endowed with distinct chemical, mechanical, optical, and physical properties as a result of their structural characteristics, such as high porosity, low density, renewability, and biodegradability. Unmodified and modified chitin and chitosan, as well as their various derivatives, are used in applications such as dye adsorption [145], air pollution [146], and heavy metal adsorption [147]. However, their main disadvantages include their low absorption capacity, poor mechanochemical stability, and low surface area. In order to improve these features, various approaches have been experimentally adopted: imprinting with metallic species [148] or minerals [149], the modification of the biomaterial surface through the crosslinker [150], and grafting [151], resulting in more active sites that produced more reliable dye–adsorbent interactions.

### 7.4. Composites

Numerous studies on the use of natural fibers of different types (hemp [152], kenaf [153], jute [154], flax [155], curaua [156]) and architectures (short-fiber [157], non-woven mat [158], and woven fabrics [159]) as reinforcements for PLA polymer have been conducted in order to improve the biodegradability, mechanical properties, thermal properties, and flame retardancy of corresponding composites [160]. By interlacing 3D-braided yarn produced by the solid braiding method, a plain woven fabric in flax material was produced. The obtained system was then combined with PLA polymer to create a sheet using the solution casting technique. Finally, composite laminates were prepared by film stacking and compression molding, and their tensile, flexural, and impact properties were studied in relation to the number of layers of fabric and loading along the warp and weft directions. The final results confirmed that fax fibers worked as effective reinforcing agents for the PLA polymer, improving its thermal and mechanical properties [161].

Polymers and composites can absorb varying amounts of water depending on their chemical nature, formulation, and environmental conditions of humidity and temperature [162]. Composite applications, ranging from civil structures to medical implants, necessitate long-term studies in moist environments [163]. The dominant mechanism in the phenomenon of moisture penetration is the diffusion of water molecules into the matrix, and also into the fibers, which is enabled by a capillary flow along the fiber–matrix interface, followed by diffusion from the interface into the bulk resin and transport via micro cracks [164]. Often, the surface damage and cracks caused by absorption facilitate the entry of water into the composite [165]. Liquid swelling is an important experiment to understand the composites’ performance in wet environments. The mechanical and swelling behavior of a fully biodegradable “green” textile composite made from Ecoflex polymer and ramie fabric by using the hot compression molding technique was analyzed in the work of Kumar et al. [166]. The tensile strength, tensile modulus, elongation at break, and diffusion characteristics of the composites in water, naphthenic oil, and diesel were measured. From the values, the tensile strength and Young’s modulus of Ecoflex/ramie mat composite were more than that of the neat polymer. The mechanism of the diffusion followed the classical Fick law of mass transport with good approximation. The polar molecules of water easily penetrated in the cellulosic polar fiber (ramie); thus, the Ecoflex/ramie fabric composite absorbed more water than diesel and lubricating oil. 

### 7.5. Personal Thermal Management

**Figure 8:**
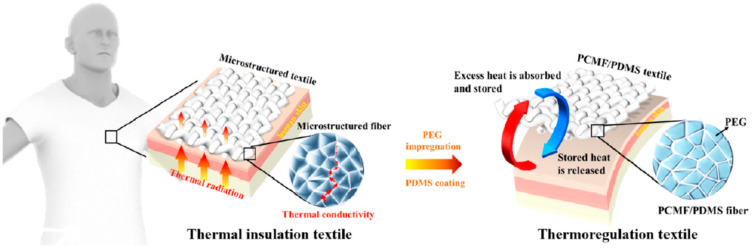
Schematization of thermal insulation textile made from microstructure fibers impregnated with biocompatible PEG and coated with PDMS. Reprinted [167].

Personal thermal management is currently receiving significant attention and interest because it can keep people comfortable while saving energy at the same time. This technology aims to heat or cool the human body locally, without wasting energy used for heating, ventilation, and air conditioning. Nonetheless, previous studies highlighted weak points, such as limited working temperature, poor comfort, and low textile reliability. In a study by Wu et al. [167], a skin-friendly personal insulation textile and a thermoregulation textile capable of performing both passive heating and cooling with a single piece of textile and zero energy input was developed. A freeze-spinning process was used to create a micro-structured biomaterial from breathable and antibacterial silk fibroin, resulting in good thermal insulation, low thermal emissivity, and good dyeability. Next, the obtained microstructure fibers were filled with biocompatible phase-change materials (poly(ethylene glycol), PEG) through the impregnation and coated with polydimethylsiloxane (PDMS) to enhance the hydrophobic and mechanical properties and prevent material leakage (Figure 8). As a result, the insulation textile was transformed into a thermoregulation textile with good water hydrophobicity, mechanical robustness, and working stability.

### 7.6. Counterfeiting Sector

Silk luminescence was achieved in the work of Zhang et al. [168] through the chemical coating of the surfaces of natural fibers with luminescent gold nanoclusters (AuNCs). The synthesis was achieved through an easy, eco-friendly, and highly reproducible method. The silk was immersed in an alkaline aqueous solution containing hydrogen tetrachloroaurate (III) hydrate (HAuCl_4_), and a redox reaction between the protein-based silk and an Au salt precursor occurred. From the experimental data, the good optical properties of the luminescent silk coated with AuNCs were established by its relatively long wavelength emission, high quantum yields, long fluorescent lifetime, and photostability. Compared to the pristine fibers, golden silk possessed superior mechanical properties, a good ability to inhibit the penetration of UV radiation, and lower toxicity in vitro. The authors proposed nanocluster-coated silk fibers as potential candidates for the commercial silk textile industry, tissue engineering, cell adhesion, antibacterial materials, biosensors and, particularly, the anti-counterfeiting sector. 

### 7.7. Hospital Clothing

With the purpose of developing disposable hospital clothing and reducing the social costs resulting from the harmful effects of pollutants, Reza Saffari et al. [169] studied the improvement of antibacterial characteristics of nonwoven biodegradable textiles, made from polylactide, by using titanium dioxide (TiO_2_) coating. The coating treatment involved a clean and environmentally benign process, i.e., the low-temperature plasma technique. Through contact with cold plasma, several concurrent processes caused chemical and physical changes to the fabric surface’s characteristics. To activate the plasma surface, gases such as oxygen, nitrogen, hydrogen, and ammonia were used. The interaction of these gases with the surface resulted in the formation of various chemical functional groups. The solvents, surfactants, and drying ovens typically used in the pretreatment and finishing of textile fabrics are not required in the case of plasma, by making this coating technique clean and environmental safety. The final results made it possible to determine a reduction in both bacteria, *S. aureus* and E. coli, in the treated textiles as a function of the coating processing time.

## 8. Introducing Bio-Sustainable Textile Materials to the Market

In 2018, Aquafil and Genomatica announced a multi-year collaboration to create a commercially viable bioprocess for producing caprolactam from plant-based renewable ingredients. This process was applied to the production of 100% sustainable nylon by combining the skills of the first (Aquafil) in the production of polyamide 6 with those of the second (Genomatica) process technologies to produce chemicals from alternative feedstocks in a more cost-effective, sustainable, and performance-oriented manner [170].

In 2019, in Trentino (Italy), a vegan and coated fabric named VEGEA was developed by a company of the same name. The name originated from the union of VEG (Vegan) and GEA (Mother Earth). It was chosen as an alternative material totally based on oil- and animal-derived sources, characterized by a high content of vegetal/recycled raw materials such as vegetal oils and natural fibers from agroindustry. In fact, VEGEA is a plant-based technical fabric, obtained from the treatment of the fibers and oils of marc, a natural derivative of wine production, including grape skins, seeds and stalks. Its production can also be considered entirely sustainable and “vegan-friendly”), since it does not use oil or pollutants, and does not consume water or animal derivatives [171].

The North Face and Spiber released the “Moon Parka” in 2019. This silk-like yarn was made from bio-fabricated brewed proteins and produced through a fermentation process involving sugars and microbes. Vivo Barefoot developed a bio-based vegan version of their popular performance shoes, the Primus Lite, made with 30% biobased materials. Flexible and stretchy characteristics were produced by a combination of an algae-based natural foam, Vietnamese natural rubber, and biosynthetic material derived from corn [172].

Eastman and DuPont Biomaterials promoted a fabric collection made from sustainable, bio-based materials by combining Eastman NaiaTM and DuPontTM Sorona fibers to create garments with exceptional stretch and recovery, luxurious drape, and a smooth, soft feel (September 2020). Eastman Naia is a cellulosic fiber applied in womenswear, whereas DuPont™ Sorona is a high-performance polymer certified as a bio-based product. In June 2021, a new fabric collection made with sustainable, bio-based materials was launched by Dupont and JayaShree Textiles [173].

DuPont Biomaterials and Welspun India announced a new, bio-based home textile collection, including bath towels and bedsheets (October 2021). Bolt Threads, a material solutions company, created a revolutionary, certified bio-material, called Mylo, by drawing inspiration from nature. It is created by engineering mycelium, which is composed primarily of renewable natural ingredients. It is marketed as a sustainable alternative to leather that will be made available to the public in 2022 by a consortium of companies (Adidas, Kering, Lululemon, and Stella McCartney) [174].

## 9. Conclusions

This work offers an overview of recent potential applications of bio-materials in the textile industry. The majority of textile fibers used around the world are made from petroleum-derived plastics (polyester, polyamide, polypropylene). However, in addition to all the benefits of fossil-based plastics (such as their light weight, low cost, durability, and chemical resistance), the negative environmental consequences of their production and uses have recently received significant attention. Textile production is one of the most pollutive industries due to its use of hazardous chemicals, consumption of water and energy, and high gas emissions. Other sources of contamination arise from the end-of-life of these products due to waste accumulation in nature, in oceans, or in landfills, or due to further gas discharges during incineration. Given the significant contamination of our ecosystem caused by synthetic fibers involved in the textile industry, biomaterials derived from renewable resources or endowed with biodegradability characteristics have been proposed as a possible green solution for reducing the environmental impact of fabric production. The use of polymers derived from renewable sources (both biodegradable and non-biodegradable) would result in reduced greenhouses emissions (GHG) and fossil fuel consumption (FFC) when compared to common fossil-based, non-biodegradable polymers. Although less biodegradable compared to natural-based fibers (wool, cotton), aliphatic polyester bio-based fibers are biodegraded more quickly compared to PET fibers. Furthermore, the larger moisture vapor transmission of bio-based polymers compared to PET, nylon and PP materials, allows greater breathability by corresponding fabrics. Bio-based fibers are also endowed with good mechanical resistance and antibacterial properties. Research studies confirmed the applicability of biopolymers in blend formulations to the production of antibacterial fabrics, to increasing fabric resistance against harmful UV radiation, to the thermoregulation of passive heating and cooling, to composites, or in the anti-counterfeiting sector. The application of biopolymers was also found to be useful in the pre-treatment or finishing of fabrics, in 3D printing to replace traditional printing methods involving hazards chemicals, and in the purification of dye-contaminant water. Finally, several suggestions from major brands to shift production toward environmentally friendly materials and green technologies were also presented.

## Figures and Tables

**Table 1:** Main properties of common bio-based and traditional plastic materials.

	Monomers and Sources	Tensile Strength [MPa]	Biodegradability		Water Vapor Permeability (g·mil/m^2^·Day·kPa)	Density (g/cm^3^)
			Environment	Biodegradability (%)		
PLA	Lactide acid from starch or sugar cane corn stover, sugarcane bagasse, rice hulls, woody crops and forest residues [34]	37−66 (at yield) [49]	Compost (30 days)	60–70 [49]	400 [50]	1.21–1.25 [51]
		61.6–49.6 [52]	Soil (120 days)	0 [49]		
		21–60 [51]	Marine (180 days)	3–4 [49]		
PHA	Hydroxyalkanoates accumulated inside the cells of microorganisms [28]	17–104 [52]	Compost (180 days)	94 [53]	/	1.25 [54]
			Soil (90 days)	100 [53]		
			Marine (365 days)	52–82 [53]		
PHBV	Hydroxybutyric monomers and hydroxyvalerate from carbon sources [36]	20−40 (at yield) [49]	Compost (28 days)	80 [49]	30 [50]	1.18–1.262 [51]
		40 [51]	Soil (280 days)	48.5 [49]		
			Marine (180 days)	38–45 [49]		
PCL	6-hydroxycaproic acid, ε-caprolactone [37]	25−33 (at yield) [49]	Compost (91 days)	100 [55]	200 [50]	1.11–1.146 [51]
		20.7–4 [51]	Soil (280 days)	50 [55]		
			Marine (365 days)	30 [56]		
PBAT	Butanediol (BDO), adipic acid (AA) and terephthalic acid (PTA) [38]	13−15 (at yield) [49]	Compost (45 days)	34–67 [49]	1200 [50]	1.26 [57]
			Soil (120 days)	6.6 [49]		
			Marine (28 days)	1–1.4 [49]		
PBS	Succinic acid from glucose, starch, xylose, or oil sources [39]	30−35 (at yield) [49]	Compost (160 days)	90 [49]	90 [50]	1.26 [58]
		24.8 [52]	Soil (28 days)	1 [49]		
			Marine (28 days)	1 [49]		
Bio-PE	Ethanol from sugar [42]	Similar to petroleum-based [49] 17.9–33.1 [52]	Very slow [52]/Not biodegradable [59]		3 [50]	Similar to petroleum-based [60]
Bio-PP	Methanol, isopropanol, ethanol, butanol, and glycerin from biomass [43]	29.3–38.6 [52]	Very slow [52]		/	Similar to petroleum-based [60]
Bio-PET	Terephthalic acid and ethylene glycol from biomass (forest residues, corn stover) [44]	Similar to petroleum-based [49] 50 [52]	Very slow [52]/Not biodegradable [59]		/	Similar to petroleum-based [60]
Bio-PA	11-aminoundecanoic acid, 1,8-octanedicarboxylic acid, 1,10-decanediamine) from castor oil [45]	37−41 (at yield) [49]	Not biodegradable		/	Similar to petroleum-based [60]
PE	Ethylene from fossil sources	10–32 (at yield) [49]	Not biodegradable		1.7–8.7 [61]	1.3–1.8 [60]
PP	Propylene from fossil sources	15–27.5 (at yield) [49]	Not biodegradable		4 [50]	~0.95 [60]
PET	Terephthalic acid from fossil sources	55−79 (at yield) [49]	Not biodegradable		20 [50]	1.33–1.48 [60]
PA	Caprolactam(nylon–6), Adipic acid (nylon–6,6) from fossil sources	49–69 [61]	Not biodegradable		350 [50]	1.07–1.24 [60]

**Table 2:** Main properties of most common natural, synthetic, and bio-based fibers.

	Yarn Production Process	Tenacity (cN/dtex)	Melting Point (°C)	Moisture Absorption	
				Moisture Regain (%) -	Overall Moisture Management Capacity
PLA	Melt spinning at 210 to 235 °C [93]	0.90–1.09 [93] 4–5.5 [114] 3.2–5.5 [115]	162.4–162.7 [93] 175–180 [114]	0.5 [114] 0.4–0.6 [115]	/
PLA/PHBV 90/10 (*w/w*)	Melt spinning at 210 to 230 °C [93]	0.46–1.09 [93]	164.3–164.9 [93]	/	/
PLA/PHBV 80/20 (*w/w*)	Melt spinning at 210 to 230 °C [93]	0.59–0.64 [93]	164.4–164.8 [93]	/	/
Cotton	/	1.8 [116] 3.1 [117] 1.9–3.1 [115]	/	7.5 [88] 8.5 [115]	0.63 [116]
Viscose	/	2 [116]	/	/	0.62 [116]
Viscose/Modal 50/50	Ring spinning technique [116]	2 [116]	/	/	0.6 [116]
Viscose/Cotton 50/50	Ring spinning technique [116]	1.5 [116]	/	/	0.64 [116]
Cotton/Silk (85/15)	Ring and siro spinning systems [117]	1.6–2.2 [117]	/	/	0.58–0.62 [117]
Cotton/Silk (55/45)	Ring and siro spinning systems [117]	2.42–2.52 [117]	/	/	0.57–0.66 [117]
Wool	/	1–1.4 [115]	/	14–18 [88] 14.5 [115]	/
Silk	/	4.7 [117] 1.9–5.1 [115]	/	10.5 [118] 30 [117]	/
Polyester (PET)	Melt spinning at 280–290 °C [114]	3.5–5 [114] 5.6 [119]	265 [114]	0.2–0.4 [88] 0.4 [114]	/
Polyamide	Melt spinning	3.5–5 [114] 6.6 [119]	214 [114]	4.1 [88] 4.5 [114]	/
Polypropylene	Melt spinning	3.5–5 [114] 6.5 [119]	175 [114]	0 [114]	/

## Data Availability

The data presented in this study are available on request from the corresponding author.
